# The Enzymatic Core of Scorpion Venoms

**DOI:** 10.3390/toxins14040248

**Published:** 2022-03-31

**Authors:** Gustavo Delgado-Prudencio, Jimena I. Cid-Uribe, J. Alejandro Morales, Lourival D. Possani, Ernesto Ortiz, Teresa Romero-Gutiérrez

**Affiliations:** 1 Departamento de Medicina Molecular y Bioprocesos, Instituto de Biotecnología, Universidad Nacional Autónoma de México, Cuernavaca 62210, Mexico; gustavo.delgado@ibt.unam.mx (G.D.-P.); jimena.cid@ibt.unam.mx (J.I.C.-U.); possani@ibt.unam.mx (L.D.P.); 2 Traslational Bioengineering Department, Exact Sciences and Engineering University Center, Universidad de Guadalajara, Guadalajara 44430, Mexico; jalejandro.morales@academicos.udg.mx

**Keywords:** enzymatic core, proteomics, scorpion venom, transcriptomics, venom enzyme

## Abstract

Enzymes are an integral part of animal venoms. Unlike snakes, in which enzymes play a primary role in envenomation, in scorpions, their function appears to be ancillary in most species. Due to this, studies on the diversity of scorpion venom components have focused primarily on the peptides responsible for envenomation (toxins) and a few others (e.g., antimicrobials), while enzymes have been overlooked. In this work, a comprehensive study on enzyme diversity in scorpion venoms was performed by transcriptomic and proteomic techniques. Enzymes of 63 different EC types were found, belonging to 330 orthogroups. Of them, 24 ECs conform the scorpion venom enzymatic core, since they were determined to be present in all the studied scorpion species. Transferases and lyases are reported for the first time. Novel enzymes, which can play different roles in the venom, including direct toxicity, as venom spreading factors, activators of venom components, venom preservatives, or in prey pre-digestion, were described and annotated. The expression profile for transcripts coding for venom enzymes was analyzed, and shown to be similar among the studied species, while being significantly different from their expression pattern outside the telson.

## 1. Introduction

Scorpion venoms are complex mixtures of more than a hundred components, including peptides, proteins, lipids, heterocyclic compounds, carbohydrates, etc., of which only a few have been studied in depth [1]. Peptide toxins acting on different ion channels, together with other short peptides (such as non-disulfide-bridged peptides, NDBPs), are thus far the best characterized components [2]. In this complex biofluid, other less studied components play relevant biological roles. Such is the case of the enzymes, which participate in various processes, including post-translational modification of polypeptide precursors [3], spreading of other venom components [4], and as toxic agents themselves, causing envenomation-related effects [5]. For instance, hyaluronidases are common in animal venoms, including those of spiders, snakes, and scorpions [6]. They play an important role in envenoming by facilitating the diffusion of venom components at the site of the sting/bite [7]. There are other notable examples, such as sphingomyelinase D, identified in the venom of the scorpion *Hemiscorpius lepturus* [5] and previously reported in the brown recluse spider *Loxoceles reclusa*. This enzyme is cytotoxic and causes dermonecrosis [8], and in the case of the scorpion sting, can lead to severe nephrotoxic, hepatotoxic and hemolytic complications [9]. Antareases, which are Zn-metalloproteinases, are ubiquitous in scorpion venoms [10]. They may cause pancreatitis in stung patients via a proposed intracellular mechanism [11], a pathophysiological effect of scorpion envenoming that is often overlooked.

Enzymes from animal venoms have a wide spectrum of potential applications, as exemplified by snake venom enzymes or their derivatives that have been applied as medical treatments and diagnostic tools [12]. Aggrastat^®^ (Tirofiban) and Integrilin^®^ (Eptifibatide), two drugs based on snake venom disintegrins, are available on the market as antiplatelet agents. Tirofiban was developed based on the sequence of snake venom disintegrins isolated from the venom of the Indian saw-scaled viper (*Echis carinatus)*. Eptifibatide is a peptide designed to mimic a small portion of barbourin, a disintegrin found in the venom of the Southeastern pygmy rattlesnake (*Sistrurus miliarus barbouri*) [13]. Defibrase© and Reptilase^®^ (Batroxobin) are thrombin-like serine proteases isolated from the venoms of the Brazilian (*Bothrops moojeni*) and common (*Bothrops atrox*) lanceheads, respectively. Defibrase© functions as a fibrinolytic agent and is used in patients with thrombosis. Reptilase^®^ is used as a hemostatic drug, and, in medical laboratories, to measure fibrinogen levels and blood coagulation capability, as well as for the detection of dysfibrinogenemias [14]. Protac^®^ is a fast-acting protein C activator isolated from the venom of the copperhead snake *Agkistrodon contortrix*. It is a potential antithrombotic agent and a tool for the preparation of activated protein C, and is also used for the diagnosis of disorders in the protein C pathway [15].

In recent years, the development of technologies in the fields of genomics, proteomics and transcriptomics has increased the information available on venom components by orders of magnitude, which favors the discovery of more candidates to be considered for pharmacological and industrial use. These methodologies have confirmed that venomous animals are prolific sources of enzymes and other components, highlighting the importance of their identification and characterization. Enzymes from venoms of animals other than snakes have been studied to a limited extent, and their biotechnological potential has not been exploited. Other venomous taxa, such as arthropods, have also been shown to possess an enzymatic arsenal in their venoms. In this work, the diversity of enzymes present in the venom of several scorpion species is explored, using the sequences obtained from transcriptomic analyses of venom glands and proteomic analyses of venoms. The analyses were performed using previously reported transcriptomic and proteomic data (Table 1), covering Old World and New World scorpions. Sampling included species from family Buthidae, the largest scorpion family, widespread around the world and with several species of medical importance [16]; family Urodacidae, scorpions of the Old World [17]; family Vaejovidae, from North and Central America, the one with the largest diversity of species in Mexico and with a wide array of components with biotechnological potential in the venom [18]; family Euscorpiidae, widespread in Central and Southern Europe, the Mediterranean coast of Africa, and the Americas [19]; family Hadruridae, recently elevated from subfamily status [20], comprised of large scorpions from USA and Mexico, and family Superstitionidae, which includes a single genus with one extant species [21].

## 2. Results

### 2.1. Transcriptomic Analysis

The origin of the used transcriptomic data is heterogeneous, therefore there is no homogeneous sequencing depth among the samples. Despite this, the results obtained in the analyses showed a similar trend in the profile of identified enzymes. The median of the sequencing depth among all studied transcriptomes was over 75 million reads, and contig assembly resulted in a median number of transcripts of ca. 131,000 (Supplementary Table S1).

Previous venom gland transcriptomic analyses which focused on the identification of transcripts related to venom components, have mainly identified enzymes annotated as hydrolases, oxidoreductases and transferases (Table 1). The presence of these types of enzymes in the venom has also been confirmed in some of these studies by proteomic analysis. Among the hydrolases in particular, phospholipases, hyaluronidases and proteases have been identified (Table 1).

### 2.2. Putative Enzyme Identification, Orthogroup Assignment and Annotation

A total of 4145 sequences potentially coding for enzymes were identified, which were clustered into 330 orthogroups belonging to 4 of the 7 major EC classes (the EC code indicates the Enzyme Commission number) defined by the Nomenclature Committee of the International Union of Biochemistry and Molecular Biology (NC-IUBMB) (https://www.qmul.ac.uk/sbcs/iubmb/enzyme/ accessed on 11 October 2021). Of these, 65 orthogroups corresponded to oxidoreductases (EC1), 67 to transferases (EC2), 188 to hydrolases (EC3) and 10 to lyases (EC4).

**Figure 1:**
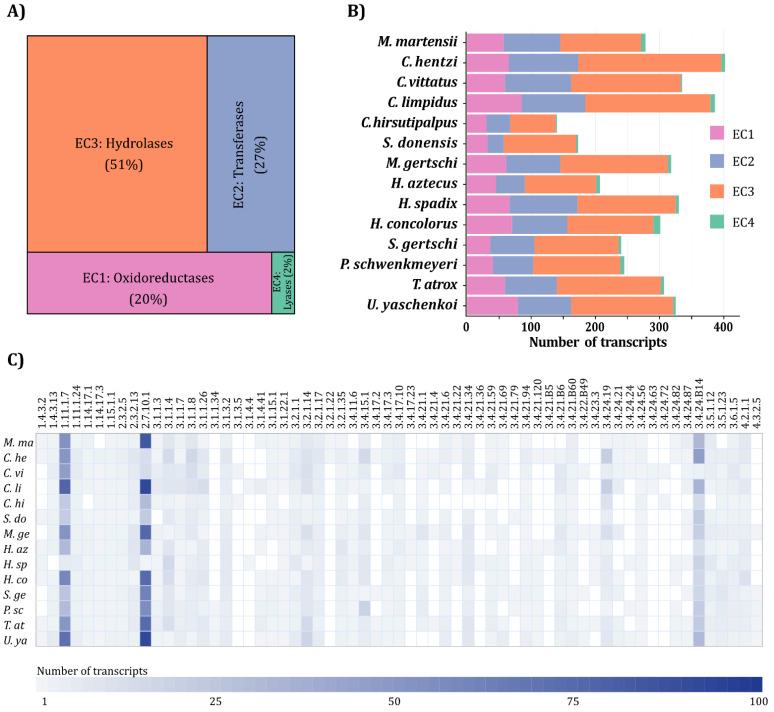
Classification of putative enzymes derived from scorpion venom gland transcriptomes: (**A**) Tree map showing the relative diversity of recovered enzyme-coding transcripts, grouped by enzyme class; (**B**) Bar plot showing the total number of the different enzyme-coding transcripts per scorpion species; (**C**) Heatmap of the different ECs identified in this study and the number of transcripts per species. In (**A**,**B**), colors identify transcripts coding for enzymes of the following EC classes: ■ oxidoreductases (EC1), ■ transferases (EC2), ■ hydrolases (EC3), ■ lyases (EC4). In (**C**), the blue gradient indicates the number of transcripts annotated per EC identifier.

Transcripts were assigned to a global classification scheme of unique 63 ECs (Supplementary Table S2). The most diverse enzymes were hydrolases (EC3) with 2124 coding sequences, followed by transferases (EC2) with 1125 coding sequences. Less diverse were oxidoreductases (EC1) with 821 sequences, and lyases (EC4) with 75 sequences (Figure 1A).

In general, scorpions from family Buthidae presented the largest diversity of putative enzymes. In particular, *C. hentzi*, was the richest species, with 69 oxidoreductases, 115 transferases, 232 hydrolases and 7 lyases, despite not having the transcriptome with the greatest sequencing depth (Figure 1B, Supplementary Table S1). At the opposite end of the diversity spectrum, for *C. hirsutipalpus*, the same pattern of enzyme diversity was observed as for the rest of the species, however, the number of recovered sequences was significantly reduced due to the low sequencing depth (Supplementary Table S1).

**Figure 2:**
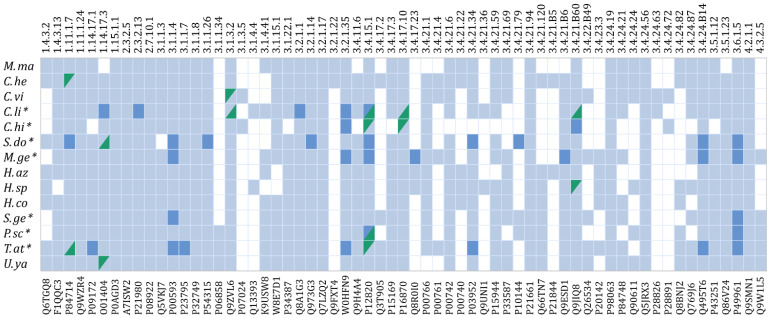
Presence/absence profile of enzyme-coding transcripts in transcriptomes and enzymes in proteomes, per species. Translated sequences from each transcriptome were cross-matched against the seven available proteomes. An asterisk next to the species name indicates that the proteome is available and was analyzed. Boxes in light blue identify sequences found only in the transcriptome, and boxes in dark blue identify sequences found in both the transcriptome and the proteome of the same species. Several transcripts matched heterologously with venom proteins from a different species, and are indicated in split boxes in green. A box with a green upper left side specifies the transcript and a box with a green lower right side in the same column indicates the matching protein (for EC 3.4.15.1, the cognate pairs are between buthids (*C. hirsutipalpus* and *C. limpidus*) and between vaejovids (*T. atrox* y *P. schwenkmeyeri*)). In the upper part of the graph, the ECs of the enzymes are indicated, and in the lower part, the corresponding UniProt IDs are given.

Oxidoreductases and hydrolases are the most diverse enzyme classes in all scorpions, regardless of their taxonomic family, while transferases and lyases are the least diverse (Figure 2).

### 2.3. Transcript Sequence Validation by Proteomic Matching

The enzyme-coding sequences derived from the transcriptomic analyses were used as input database for the identification of enzyme fragments in the available proteomic data. By this method, 57 venom enzymes were identified, among them 5 oxidoreductases, 2 transferases, and 50 hydrolases (Figure 2, Supplementary Table S3), corresponding to 22 unique ECs. As shown in Figure 2 and Supplementary Table S2, the largest number of enzymes identified in the proteomes correspond to hydrolases (EC3), followed by transferases (EC2), and oxidoreductases (EC1). A few transcripts from species with no available proteome matched proteins heterologously from the venoms of other species (Figure 2).

Regarding the annotation of enzymes detected in the venom, the presence of 9 ECs previously identified in scorpions was corroborated, including peptidylglycine monooxygenase (PHM) (1.14.17.3), dopamine beta monooxygenase (1.14.17.1), phospholipase A2 (3.1.1.4), chitinase (3.2.1.14), hyaluronidase or hyaluronoglucosaminidase (3.2.1.35), peptidyl-dipeptidase A (3.4.15.1), neprilysin-2 (3.4.24.B14), acetylcholinesterase (3.1.1.7) and alpha-amylase (3.2.1.1). In addition, 7 new ECs were identified for enzymes not previously described in scorpion venoms, though present in other animal venoms. These include peroxidase (1.11.1.7), protein-glutamine gamma-glutamyltransferase (2.3.2.13), acid phosphatase (3.1.3.2), angiotensin-converting enzyme 2 (3.4.17.23), apyrase (3.6.1.5), granzyme B (3.4.21.79), and epitheliasin (3.4.21.B60). A few enzymes not previously reported in other venomous animals were found, including galactolipase (3.1.1.26), carboxypeptidase E (3.4.17.10), plasma kallikrein (3.4.21.34) and prostasin (3.4.21.B6).

### 2.4. Novel Enzymes

**Figure 3:**
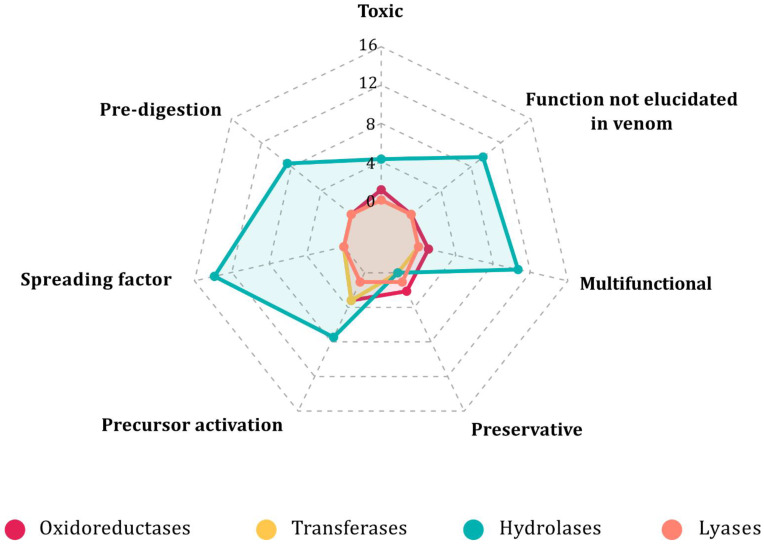
Distribution of enzyme classes according to their physiological function. Counts denote the number of ECs per category.

The enzymes with transcripts identified in the transcriptomes and corroborated in the proteomes (Supplementary Table S2) have different biological activities. For simplification purposes, 7 global functions were assigned to the 63 identified ECs. First, information on the enzyme’s involvement in the physiological effects of the venom was considered when available. Then, the information from other physiological phenomena, not necessarily reported for venoms, but related to their enzymatic activities and their possible role in envenomation was also included. The analysis showed that hydrolases are involved in almost all functions excluding the “Preservative” category. It is noteworthy that only hydrolases are found in the “Pre-digestion”, “Spreading factor” and “Multifunctional” categories. By contrast, members of all four classes identified in the transcriptomes were found in the “Precursor activation” category (Figure 3).

#### 2.4.1. Toxic Enzymes

One of the most relevant effects of the enzymatic components of animal venoms is related to their direct toxicity or to the harmful physiological effects triggered by the products of their catalysis. Transcripts for several enzymes associated with neurotoxicity were identified in the analysis, such as acetylcholinesterase (3.1.1.7) and cholinesterase (3.1.1.8), and with cytotoxic effects such as L-amino-acid oxidase (1.4.3.2), phospholipase D (3.1.4.4) and sphingomyelin phosphodiesterase D (3.1.4.41). Of them, the presence of acetylcholinesterase in the venom was corroborated by proteomic analysis (Supplementary Table S2).

#### 2.4.2. Enzymes Involved in Prey Pre-Digestion

Venom enzymes contribute to prey pre-digestion. Hydrolases are frequently associated with this physiological function, as they are responsible for breaking the bonds of larger biomolecules to form units that can be assimilated by the digestive system [33]. Eight hydrolase subclasses were identified in the transcriptomes, corresponding to galactolipase (3.1.1.26), triacylglycerol lipase (3.1.1.3), alpha-amylase (3.2.1.1), chitinase (3.2.1.14), alpha-galactosidase (3.2.1.22), chymotrypsin (3.4.21.1), cathepsin L1 (3.4.22.B49) and ceramidase (3.5.1.23). Alpha-amylase and chitinase were also confirmed by proteomic analysis (Supplementary Table S2). Since the basis of scorpions’ diet consists mainly of arthropods, animals with chitin exoskeletons [34], it is not surprising that chitinase was found in the venom.

#### 2.4.3. Enzymes as Spreading Factors

Enzymes with activities that compromise tissue integrity and promote the dissemination of venom components have been described in other venomous animals. These enzymes are therefore called dispersion or spreading factors [7]. Sequences putatively coding for eight enzymes related to this function were identified exclusively by the transcriptomic analysis. These are deoxyribonuclease II (3.1.22.1), 5′-nucleotidase (3.1.3.5), protein C (activated) (3.4.21.69), coagulation factor IXa (3.4.21.22), coagulation factor Xa (3.4.21.6), fibrolase (3.4.24.72), ADAMTS-4 endopeptidase (3.4.24.82) and ADAMTS13 endopeptidase (3.4.24.87). Additionally, five other enzymes were identified by the sequences of the precursors in the transcriptomes and corroborated in the proteomes, including hyaluronoglucosaminidase (3.2.1.35), peptidyl-dipeptidase A (3.4.15.1), angiotensin-converting enzyme 2 (3.4.17.23), plasma kallikrein (3.4.21.34) and granzyme B (3.4.21.79).

#### 2.4.4. Enzymes as Venom Component Activators

Transcriptomic analysis identified 14 ECs with functions related to the activation of polypeptide precursors. They can be grouped into two categories: enzymes with proteolytic activity, and enzymes with biomolecule-modifying activity. To the former group belong lysozyme (3.2.1.17), aminopeptidase B (3.4.11.6), oviductin (3.4.21.120), lysine carboxypeptidase (3.4.17.3), carboxypeptidase E (3.4.17.10), proprotein convertase 2 (3.4.21.94), peptidyl-alpha-hydroxyglycine alpha-amidating lyase (4.3.2.5), of which only carboxypeptidase E was detected in the proteome. To the latter, protein-lysine 6-oxidase (1.4.3.13), glutaminyl-peptide cyclotransferase (2.3.2.5), biotinidase (3.5.1.12), receptor protein-tyrosine kinase (2.7.10.1), protein-glutamine gamma-glutamyltransferase (2.3.2.13), dopamine beta-monooxygenase (1.14.17.1) and peptidylglycine monooxygenase (1.14.17.3), with the last three also corroborated in the venom.

#### 2.4.5. Enzymes with Preservative Function

The enzymes superoxide dismutase (1.15.1.1), carbonic anhydrase (4.2.1.1) and peroxidase (1.11.1.7) were identified from transcripts, the latter being corroborated in the proteome. The function of these proteins is related to the elimination of reactive oxygen species (ROS), which are intermediates or end products of cellular metabolism [35]. Venom components are known to be susceptible to ROS which reduce their half-life [36].

#### 2.4.6. Multifunctional Enzymes

The enzymes included in this category are associated with multiple functions in the venom. They may act as dispersing factors, precursor activators, toxic components, allergens and/or pre-digestive agents.

Transcripts putatively coding for thioredoxin-dependent peroxiredoxin or peroxiredoxin-4 (1.11.1.24), lipoprotein lipase (3.1.1.34), venom exonuclease 1 (3.1.15.1), carboxypeptidase B (3.4.17.2), trypsin (3.4.21.4), astacin (3.4.24.21), gelatinase A (3.4.24.24), neprilysin-2 (3.4.24.B14), phospholipase A2 (3.1.1.4), acid phosphatase (3.1.3.2) and apyrase (3.6.1.5) were found. Of them, the last four were detected in the venom.

#### 2.4.7. Enzymes with Non-Elucidated Function in Venom

The enzymes assigned to this category have no previous reports of venom-related activity. Additionally, no direct connection between their reported activity and the pathophysiology of venoms was identified. However, they meet the criteria to be considered enzymes that can be secreted by the venom gland and could end up in venom (see Section 5). Transcriptomic analysis revealed transcripts for enzymes of this kind, belonging to 9 ECs, corresponding to tryptase (3.4.21.59), procollagen C-endopeptidase (3.4.24.19), insulysin (3.4.24.56), meprin B (3.4.24.63), mast cell protease 5 or chymase 2 (3.4.21.B5), pancreatic elastase (3.4.21.36), gastricsin (3.4.23.3), prostasin (3.4.21.B6) and epitheliasin (3.4.21.B60). The confirmation of the latter two in the proteomes demonstrates that, though their function in the venom has not been established, enzymes in this group can indeed be components of the scorpion venom.

### 2.5. Determination of the Enzymatic Core

**Figure 4:**
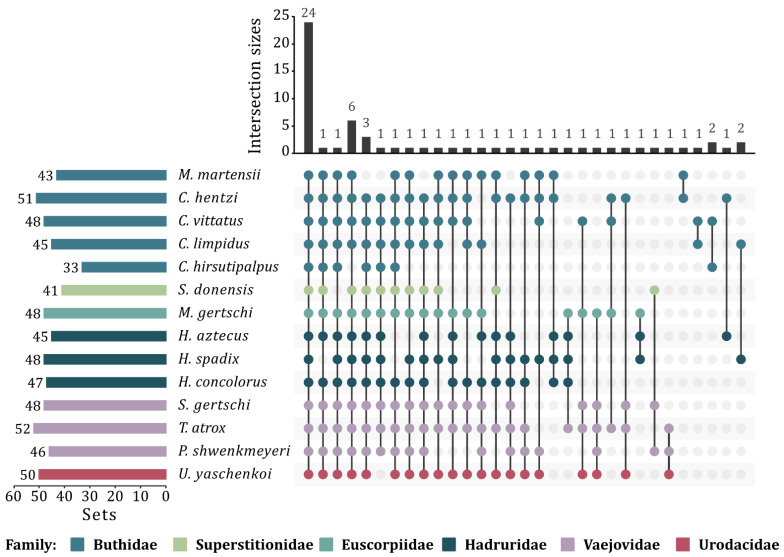
Upset plot showing the graphical representation of the enzymatic core of the venoms of the studied scorpions. The upper bar graph shows the number of ECs contained in each intersection (intersection sizes). The number of sets (left side) indicates the degree of contribution of an EC or the level of interrelationship between species. The dot plot (right side) reflects the contribution of each species to the intersections.

Transcriptomic analysis allowed the identification of enzymes which are common to all venoms of the studied scorpion families. This group of enzymes is here called the “enzymatic core” and is integrated by 24 ECs (Figure 4), with 3427 sequences cataloged into 258 orthogroups. Within these 24 ECs, 3 ECs corresponding to oxidoreductases, 3 to transferases, 17 to hydrolases and 1 to a lyase were identified (Supplementary Table S4).

Some members of the enzymatic core have already been studied. The best characterized group is that of the hydrolases, within which phospholipases, proteases and hyaluronidases have been previously described [4,5,11,37]. These enzymes have been identified in transcriptomes and proteomes. For some of them, the enzymatic activity was also characterized [38].

**Figure 5:**
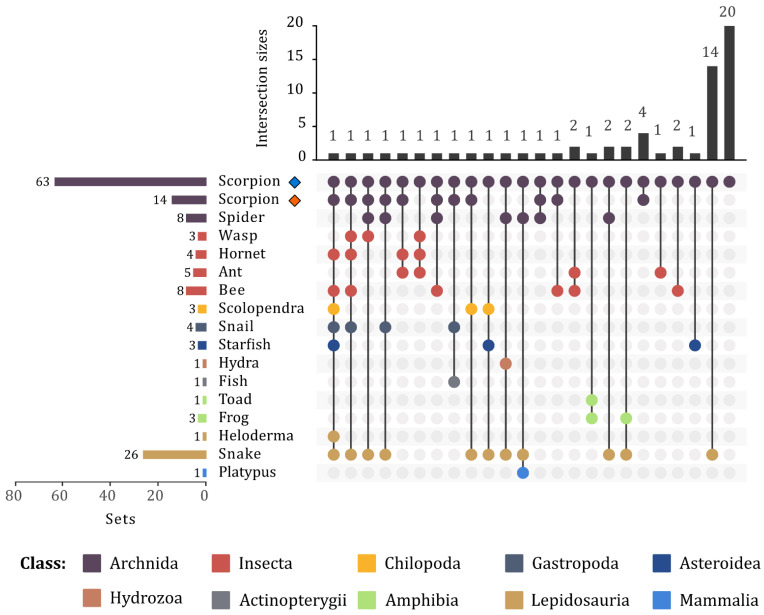
Upset plot of the enzymatic core of animal venoms. The upper bar graph shows the number of ECs contained in each intersection (intersection sizes). The number of sets (left side) indicates the degree of contribution of an EC or the level of interrelationship between each animal category. The dot plot (right side) reflects the contribution of each animal category to the intersections. Scorpion enzymes identified in this study and those obtained from existing databases are shown separately (as indicated by a blue or a red diamond, respectively).

By cross-matching all the enzymes identified in scorpions in this study with enzymes reported for other venomous taxa, it was found that 14 ECs are exclusively shared with snakes, 3 with insects (order Hymenoptera, specifically), 1 with amphibians, 2 with spiders and snakes, and 24 ECs are exclusive to the order Scorpiones. The remaining ECs are shared between two or more taxa (Supplementary Table S5). It should be noted that EC 3.1.1.4, corresponding to phospholipase A2, is the most widely distributed among animal venoms, being present in at least 8 taxonomic classes (Figure 5).

### 2.6. Venom Gland RNA-seq Quantification

**Figure 6:**
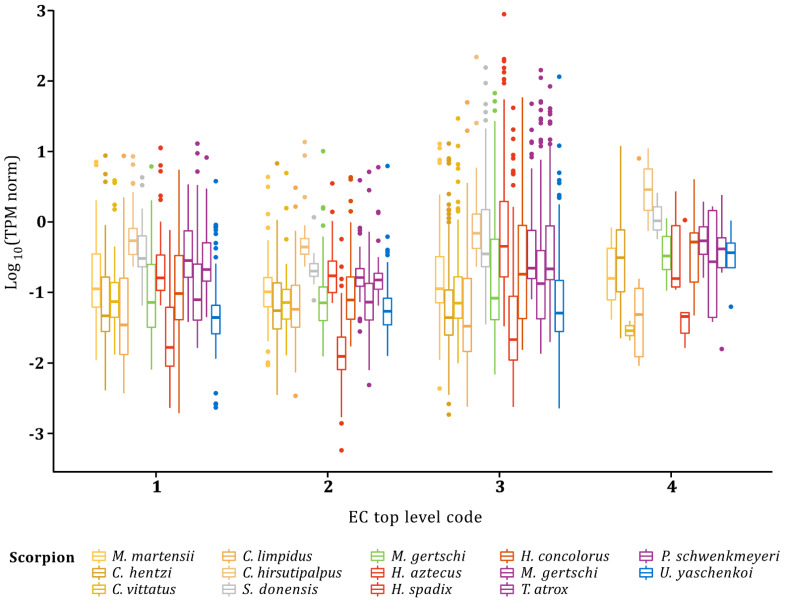
RNA-seq profile for transcripts related to enzymes in scorpion venom glands. Transcripts are grouped according to EC top level codes. Normalized transcripts per million (TPM) are shown on a Log_10_ scale. Color gamut represents different scorpion families: yellow, family Buthidae; gray, family Superstitionidae; green, family Euscorpiidae; red, family Hadruridae; purple, family Vaejovidae and blue, family Urodacidae.

The dynamics of gene expression within an organism is associated with different factors, including the nature of the tissue and its physiological state. Transcriptional and posttranscriptional regulation may result in different levels of expression, for both transcripts and mature proteins. Surprisingly, the RNA-seq profile for transcripts potentially coding for enzymes in the venom glands showed no differences between the studied species, regardless of the enzyme class, and parvorder or family of the species (Figure 6).

**Figure 7:**
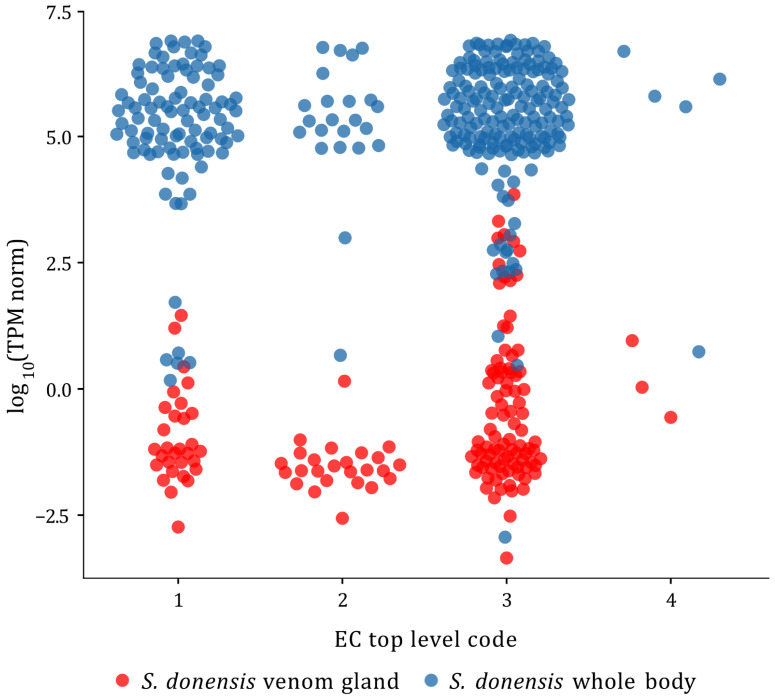
Quantitation of the transcripts coding for potentially secreted enzymes of venom gland vs. whole body in *S. donensis*. Transcripts are grouped according to EC top level codes. Normalized transcripts per million (TPM) are shown on a Log_10_ scale. Transcripts from the whole-body transcriptome are shown in blue and transcripts from the venom gland transcriptome are shown in red.

*Superstitionia donensis* is the only species for which RNA-seq has been carried out separately for the venom gland and the whole body. To determine whether the enzyme-related RNA-seq profile in the venom gland differs from that of other scorpion tissues, a comparison between these two sets from *S. donensis* was performed, considering only the sequences coding for potentially secreted enzymes. The results indicated that there is a statistically significant difference (*p* < 10^−4^) between the two transcriptomic data sets. The levels of transcript expression are higher in the body than in the venom gland (Figure 7).

## 3. Discussion

Previous transcriptomic analyses of scorpion venom glands have focused primarily on venom composition, with emphasis on peptide components, such as neurotoxins, host defense peptides and others [39]. Therefore, limited attention has been given to enzyme diversity. The purpose of this work was to identify the enzymes present in scorpion venom. Since only a few scorpion venom enzymes have been reported, data mining techniques were applied. Transcripts from venom gland transcriptomes were identified which potentially code for enzymes, according to enzyme databases of general content, such as the Braunschweig Enzyme Database (BRENDA) [40], or the ones specialized in venomous animals, such as UniProt’s Animal Toxin Annotation Project (ATAP) [41]. ATAP has 7291 entries, of which 1233 correspond to enzymes, with those from snakes (colubrids, viperids and elapids) being the most numerous with 874 sequences, as compared to only 26 scorpion enzyme sequences (https://www.uniprot.org/biocuration_project/Toxins/statistics accessed on 1 March 2021). This is understandable since in scorpions, short peptides (toxins) that alter the normal physiology of ion channels are the main agents responsible for envenomation [42], whereas in snakes, enzymes play a more prominent role [43]. Of all the identified transcripts potentially coding for enzymes, only those that could drive protein expression to secretion (i.e., contain a signal peptide-coding sequence and do not code for transmembrane domains) were selected for the analysis. The actual presence of the enzymes in the venom was validated by cross-matching the translated transcript sequences with the available scorpion venom proteomic data.

### 3.1. Transcriptomes Used in the Study

The increasing number of available transcriptomic analyses has resulted in a better understanding of the composition and function of animal venoms [44]. Although there are at least 60 scorpion transcriptomes available, many of them were obtained with the aim of reconstructing their evolutionary history [45], and therefore, either muscle tissue or the entire body were used for this objective, since no specialized tissue was required [18]. For the purposes of this work, only venom gland transcriptomes were used (with the sole exception of the whole-body transcriptome from *S. donensis* used for comparison of the expression levels).

### 3.2. Putative Enzyme Identification, Assignment and Annotation of Orthogroups

The process of describing and annotating the transcriptomes of venomous animals is typically limited to the identification of transcripts based on sequence similarity with previously reported sequences from other phylogenetically-related species, or unrelated species that share the characteristics of interest, e.g., venom production, for which experimental evidence on the presence and function of these components is available [46]. Such approach hinders the identification of novel proteins not reported in the reference species, and results in a sub-optimal exploitation of the potential offered by the large amount of sequence information generated by next generation sequencing (NGS) techniques.

Prior to this work, the enzyme classes identified in scorpions were grouped into 19 different types. This study identified 63 different enzyme types, which increases the known enzyme diversity in scorpions by 4.5 times. Besides oxidoreductases and hydrolases, the only major classes previously reported in scorpion venoms, this study demonstrates the presence of other major enzyme classes such as transferases and lyases.

To obtain an overall picture of the number and diversity of enzymes that are components of the venom and that could be shared within the species and/or families of scorpions studied, the enzymes were grouped into orthogroups sharing phylogenetic relationship, using Orthofinder, which facilitates the identification of orthologs and provides robust results exceeding those obtained by other methods, such as BLAST Reciprocal Best Hits or OrthoMCL [47,48]. Of the 330 identified orthogroups, 256 are shared between parvorders Buthida and Iurida, 34 are specific for Buthida, and 40 are exclusive to Iurida. EC 2.7.10.1 has the largest number of orthogroups, with 62, whereas 17 ECs have just one orthogroup.

As indicated in the Section 5, orthogroups that resulted in multiple EC assignments were not considered in the analysis due to conflicts with annotation. A special mention deserves the case of antareases, which were previously determined by RT-PCR to be present as venom gland transcripts in all studied scorpion species (Ortiz et al., 2014). The incomplete EC assigned to antareases in BRENDA (3.4.24.-) is shared by 123 different enzymes, of which only 43 are annotated as “secreted” in UniProt metadata, none of them being antareases. Antareases are, therefore, the kind of enzymes that could have eluded identification by the protocols used in this study, but most probably constitute part of the scorpion venom enzymatic core.

### 3.3. Validation of Transcriptome-Derived Enzymes

For a few scorpion species comprehensive, multi-omic analyses are available, including at least venom gland transcriptomics and venom proteomics. This information was used to evaluate the predicted presence of the transcriptome-derived enzymes in the venom. This is a robust strategy used to validate the biological significance of in silico reconstructions [31]. The sequences potentially coding for secreted enzymes obtained from the transcriptomic analysis were translated and used as templates for the identification of sequences generated by tandem mass spectrometry (MS/MS) of tryptic fragments from whole venom proteomic analyses. A positive match confirms that the predicted enzyme is indeed expressed, secreted and present in the venom.

In this study, 57 enzymes belonging to 22 distinct ECs were confirmed to be present in scorpion venom (Supplementary Table S3), including enzymes previously described in this venom with assessed functions, such as spreading factors [49], cytotoxic enzymes [5] or those responsible for pathologies associated with envenomation [11]. Among the enzymes correlating with those previously identified and characterized as scorpion venom components with determined activities are hydrolases, such as metalloproteases [10], hyaluronidases [50], phospholipases A2 [51] and phospholipase D (dermonecrotic sphingomyelinase) [5]. Other previously reported venom enzymes, but without assessed activities include oxidoreductases [22,24] and transferases [23].

There were three particular sequences which were not included in the reported statistics, but which deserve to be mentioned since they were found in the proteomes, and therefore confirmed to be in the venom. The transcripts encoding these enzymes did not meet the restrictions imposed on the analysis, specifically, that they should code for enzymes with a distinct EC. Two of the sequences correspond to acetylcholinesterases (3.1.1.7; 3.5.1.13) and the third to a glycosyltransferase 7 (2.4.1.133; 2.4.1.22; 2.4.1.38; 2.4.1.90). The reason why these enzymes have been assigned to more than one EC in the UniProt database was not further investigated.

It is important to note that it is not always possible to corroborate the presence in the venom of enzymes encoded by transcripts from a transcriptome. Any mechanism of expression control after transcription (including post-transcriptional, translational and post-translational regulation) can result in low protein levels that could be missed by the proteomic protocols [52]. Enzyme synthesis kinetics [53], regulation of the exocytosis pathways [54] and methodological factors, such as the selection of the enzyme that generates the fragments used for MS/MS sequencing, may hinder detection. Adding to all that, the lack of available venom proteome studies for most species results in a disparate number of sequences mined from transcriptome vs. proteomes. One relevant aspect to notice is that due to the validation scheme used in this study, in which translated enzyme-coding transcripts are used for cross-matching with the MS/MS spectra, and not the other way around, it was not possible to detect venom protein sequences when the cognate transcripts were not initially identified in the transcriptome of any of the scorpion species. The restrictions imposed on the used transcript sequences also imply that enzymes that could have ended up in the venom by endoproteolysis of membrane-bound precursors, or through a pathway different from secretion, could not be detected. This means that, besides those reported here, other enzymes could be present in the venom that could have eluded detection.

### 3.4. Novel Enzymes

**Figure 8:**
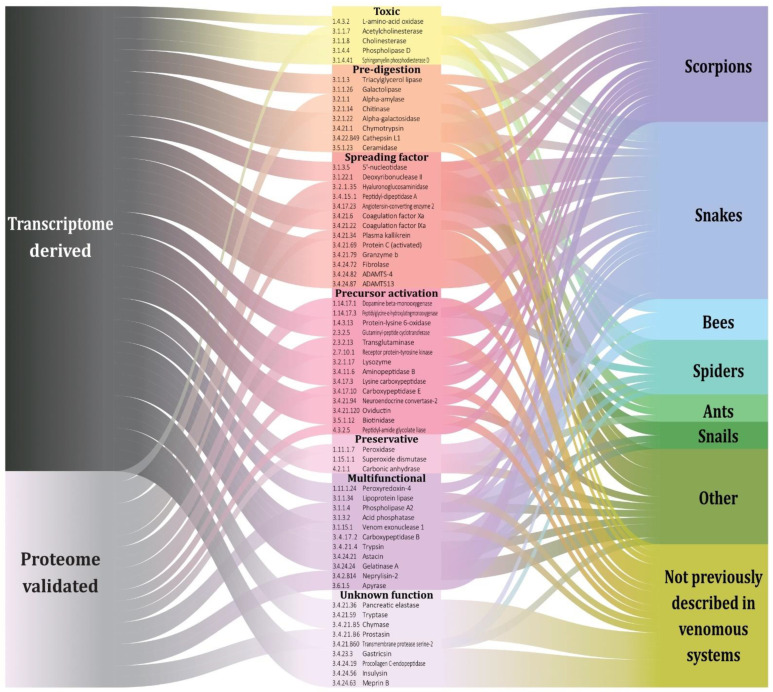
Sankey plot showing the relationship between venom enzymes identified in this study, their associated function in the venom and their presence in animal venoms. The left side segregates enzymes predicted from transcripts only, from those also validated in proteomes. At the right side, enzymes previously reported in animal venoms are shown, with the lowermost category showing novel ECs with no known counterpart in any animal venom.

Besides the identified enzymes with no previous record in scorpion venoms, but previously found in other animal venoms, where they could play a role as spreading factors [55], other novel enzymes were detected with possible different functions in the scorpion venoms. Among them, potentially toxic enzymes, precursor activators, venom component preservation factors and pre-digestive enzymes were identified. Some of the enzymes may be involved in more than one of the above-mentioned functions. A few enzymes were identified for which an apparent role in the venom could not be proposed, but which meet the criteria to be considered a venom enzyme (Figure 8).

#### 3.4.1. Toxic Enzymes

The phenomenon of scorpion sting envenoming is mainly attributed to the uncontrolled release of catecholamines, caused by alterations in the normal physiology of ion channels, due to the action of peptide toxins [42]. However, enzymatic components have been reported in scorpion venoms that may contribute to the intoxication effect. Such is the case of the cytotoxic effect associated with phospholipase D (sphingomyelinase), reported for the venom of the scorpion *H. lepturus* [5], and previously identified in recluse spiders of the genus *Loxosceles* [56]. In both, *H. lepturus* and *Loxosceles* spiders, this enzyme is the major toxic component of the venom.

Among the enzymes considered to be cytotoxic, L-amino-acid oxidase (LAAO), phospholipase D and sphingomyelin phosphodiesterase D were identified in this study at the transcriptomic level. LAAOs have been previously identified in a wide variety of venomous animals, including scorpions [57], snails [58] and snakes. In snakes, LAAOs are a major component of the venom, constituting up to 30% of the total protein content [59]. The cytotoxic effect produced by these enzymes results from the accumulation of hydrogen peroxide, a product of their catalytic activity, which induces apoptosis in the endothelial cells of blood vessels, causing hemorrhage and edema [60]. Although LAAOs from snake venoms have been studied, for other animal venoms, their function has not been demonstrated.

Enzymes were identified that in other animal venoms function as toxic components, causing tissue lesions or neurological alterations. Among the neurotoxic enzymes, acetylcholinesterase and cholinesterase were found. An interesting example of a novel scorpion venom enzyme with possible neurotoxic effects is acetylcholinesterase, identified in the transcriptomes of all studied scorpion species and corroborated by proteomics as a venom component. This enzyme has been reported in snake venoms and its main effect is to cause flaccid paralysis [61]. Although this effect is not a major symptom of scorpionism, there is evidence of scorpion sting envenoming in which patients developed flaccid paralysis [62,63,64]. Insects are part of the regular diet of scorpions, and acetylcholine is a transmitter in their central nervous system (CNS) and the target for acetylcholinesterase [65]. This cholinergic system is indispensable for insects and has even been proposed as a target for insecticide development [66]. The presence of acetylcholinesterase in the venom could contribute to the immobilization and subjugation of prey through CNS neurotransmission impairment.

#### 3.4.2. Enzymes Involved in Pre-Digestion of Prey

One of the functions attributed to venoms is to promote the degradation of prey tissues and facilitate their digestion. This function is directly attributed to the disruption of cell membranes by different mechanisms [33]. Pre-digestive enzymes facilitate the consumption of the prey by liquefying its tissues before ingestion, in a form of extra-oral digestion [67]. It is hypothesized that snake venom enzymes involved in this process are orthologues of enzymes from specific glands of the digestive system, such as pancreas and salivary glands [68], alpha-amylase being a remarkable example of this.

In this study, several hydrolases, such as lipases and ceramidases, that can act on membrane phospholipids or epicuticular lipids in insects were identified [69]. Hydrolases that degrade polysaccharides were also identified in the transcriptome and proteome, e.g., chitinase, which can degrade the chitin of the exoskeleton of arthropods, the main prey of the scorpion’s diet [70]. Protein-hydrolyzing enzymes that can degrade protein matrices of tissues, such as collagen and elastin, were identified, including chymotrypsin and cathepsin L1 [71].

#### 3.4.3. Spreading Enzymes

One of the functions by which the enzymatic fraction of animal venoms contributes to envenomation is that of favoring the passage and diffusion of venom components through tissues. Hyaluronidase is considered the diffusion factor par excellence in most animal venoms [72,73,74], including scorpions [4]. Hyaluronidase was identified in all analyzed transcriptomes, and was also detected in the venom. Other found enzymes could favor venom permeability in tissues [75]. Proteases can degrade proteins present in the extracellular matrix, such as collagen and glycoproteins [72,76] or compromise the integrity of the vascular endothelium [77], allowing venom components to enter the bloodstream. In this sense, enzymes that affect hemostasis may also aid in venom spreading by preventing the repair of blood vessels [78]. 5′-nucleotidases, ubiquitous in snake venoms, were also found in scorpion venom gland transcriptomes. In snake venoms, these enzymes have been shown to inhibit platelet aggregation due to the liberation of inhibitory AMP or adenosine by their enzymatic action [79]. It is interesting that transcripts for nucleases were also found. Nucleases can synergize with 5′-nucleotidases to produce purines, which constitute multifunctional toxins [80]. An intriguing role of nucleases in the inhibition of neutrophil extracellular traps (NETs), which can sequester venom molecules, have been suggested [81].

#### 3.4.4. Venom Component Activators

Post-translational modifications of venom components contribute to the complexity of scorpion venoms. Multiple disulfide bounds are landmarks of structurally-constrained scorpion venom peptides [82], but also C-terminal amidation, with a recently described dual system in scorpions [3] and N-terminal pyroglutamylation [83]. More than 20 types of post-translational modifications have been identified in the venom of other animals [41], among them isomerization, phosphorylation, hydroxylation and sulfurylation (sulfonation). Besides those enzyme-catalyzed chemical modifications, many venom components are expressed as polypeptide precursors which require enzymatic activation by specific proteolysis. Enzymes with both activities were found as components of the scorpion venom gland.

C-terminal amidation of toxins and NDBPs is vital for their activity [84,85,86]. It is remarkable that two components of the amidation pathway were identified in both transcriptomes and proteomes, namely carboxypeptidase E (CPE) and peptidylglycine monooxygenase (PHM). This could suggest that the peptide amidation process may continue even after peptide transition through the trans-Golgi network and secretion. The intriguing capacity of amidating enzymes to catalyze three alternative monooxygenase reactions, i.e., sulfoxidation, amine N-dealkylation and O-dealkylation [87], opens the possibility for alternative functions in the venom.

#### 3.4.5. Enzymes as Preservative Agents

It is well known that some enzymes in animal venoms produce reactive oxygen species (ROS) as byproducts, generating oxidative environments that could damage other venom components, e.g., LAAOs [88]. Enzymes that degrade ROS and therefore protect venom components from oxidation, have been identified in parasitoid wasps [89,90] and scorpions [27,91].

Superoxide dismutase, carbonic anhydrase and peroxidase were identified in the analysis. Orthologues of superoxide dismutase have been associated with protection against oxidative stress in insects [92]. Peroxidase had never been reported in animal venoms before, but in this work, its presence as both transcript in the venom gland and protein in the venom, has been demonstrated. The enzymatic activity of peroxidases may contribute to the protection and preservation of venom components from oxidative damage.

#### 3.4.6. Enzymes with Multiple Physiological Effects

Some venom enzymes can evoke multiple effects and could be considered to fall into more than one of the above categories. Enzymes of this type belong to the class of hydrolases, which can be separated into two groups, enzymes having fatty acid substrates and those acting on proteins. Among the former are phospholipase A2, lipoprotein lipase and acid phosphatase, the first two being toxic enzymes [93,94,95]. Phospholipase A2 and lipoprotein lipase also have other assignments as pre-digestive enzyme [96] and spreading factor [93,97,98], respectively. Acid phosphatase has been described as a spreading factor and an allergen [79,99]. Of the hydrolases acting on proteins, five can act as precursor activators and have a second associated function. Three of them can also act as pre-digestive enzymes (astacin, gelatinase A and trypsin) [100,101,102], one as a spreading factor (carboxypeptidase B) [103], and the last one as an adjuvant (peroxiredoxin-4) [104].

#### 3.4.7. Enzymes with Non-Elucidated Function in Venom

In this study, transcripts coding for enzymes with the necessary characteristics to be secreted by the venom gland were identified in all transcriptomes. Some of them were even corroborated by proteomics (Supplementary Table S2). However, these enzymes have not been previously described in any animal venom and it was not possible to associate their enzymatic activities with any function in the venom or effect in envenomation.

### 3.5. Scorpion Venom Enzymatic Core

Non-enzymatic components of scorpion venom, mainly toxins and other small peptides, which have been the most studied historically, are known to show specific expression profiles that could be related to the particular taxonomic family to which the species belong. It is known, for example, that venoms of scorpions from family Buthidae are richer in sodium channel-acting toxins than their counterpart from family Vaejovidae, whereas for host defense peptides, the relative abundance is the other way around [23,29].

Little is known about the expression profiles of scorpion enzymes. Biochemical characterizations have revealed the presence of three classical enzymatic activities in most scorpion venoms, corresponding to hyaluronidases, phospholipases A2 and proteases with various substrates. However, the available information concerning the presence of other enzymes was so scarce that it was impossible to establish whether there is a differential pattern in terms of enzyme diversity in the different species, nor whether there is a basal enzyme composition in all scorpion venoms.

The enzymes identified in this study have allowed the establishment of an enzymatic core with those present in all the 6 scorpion families studied. This enzymatic core consists of 24 different enzymes, including oxidoreductases, transferases, hydrolases and lyases (Supplementary Table S4). The enzymatic core will undoubtedly be expanded as more transcriptomes and proteomes are reported and the available information on scorpion venom enzymes becomes more comprehensive. The establishment of specific profiles by scorpion family was not the objective of this study, but with the information generated here, plus that which will be obtained in the future, will be possible by defining not only patterns of diversity by family, but also expression levels of the different enzymes.

Since the available information on orthologs identified in other venoms was used to make biological sense of the enzymes reported here, the question arose as to whether there is a common set of enzymes in the animal venoms described so far. Unfortunately, the information available on enzymes from most venomous animals is also limited, that it was not possible to answer this question. Although no single enzyme has been found to be present in all taxa, there is some evidence to support this possibility, at least for an important group of animals. For example, phospholipase A2 has been identified in 8 different taxa, the most widely distributed enzyme in venoms (Supplementary Table S5).

### 3.6. RNA-seq Quantification

The transcriptional profile of the different tissues of an organism varies considerably and depends, among many other conditions, on the physiological state of the cells. Venomous animals are no exception, and it has been observed, e.g., in snakes, that expression levels vary among tissues [105,106]. In scorpions, it has been shown that the transcriptional profile of the venom gland varies between the active state (after venom extraction) and the resting state (when the venom has already been replenished) [107], along with other variables, such as diet, sex [22] and ontogenetic stage [25]. Analysis of the transcriptional profiles of the 63 ECs identified in this study showed that there are no significant differences between the levels of transcripts for the different enzyme classes, regardless of the scorpion species.

Scorpion venom is produced in two venom glands located in the telson. These glands are surrounded by muscle layers and connective tissue [108]. For venom gland transcriptomic studies, the whole telson is typically dissected and processed, so the possibility of mistaking transcripts from those non-venom-related tissues for venom gland transcripts exists. As indicated above, expression patterns are usually tissue-specific. This could allow differentiation of venom gland transcripts from those of other tissues if the transcriptomes of those tissues were available as reference. This is the case for one of the species studied: *S. donensis*, for which the transcriptome of the whole body is available, as well as the transcriptome of the venom gland (i.e., the telson). RNA-seq quantification confirmed that the differences in the normalized expression levels of enzymes from both samples were significant. Remarkably, the expression levels of enzymes were lower in the telson than in the whole body. A study of the biochemical profile of regenerated venom of the scorpion *Parabuthus transvaalicus* showed that “milked” scorpions (those whose venom had been extracted) had a substantially higher (21%) mean metabolic rate than “unmilked” scorpions, up to 8 days after venom extraction. This higher metabolic rate can be linked to an increased production of precursors for venom regeneration, and therefore a higher enzymatic activity. It also showed that the high molecular weight fraction of the venom (a group of proteins of ca. 25 kDa), suggested to contain enzymes, appeared to peak on day 4 after venom extraction and waned thereafter (with their coding transcripts most probably dwindling even earlier) [109]. This is in perfect correspondence with the results reported here. For the venom gland transcriptomic analysis of *S. donensis*, the telsons were dissected 5 days after venom extraction, i.e., at a time when the overall metabolism was high and enzyme expression in the venom gland was declining. This could also explain why no significant differences were found between the expression levels of enzyme-coding transcripts in the venom glands of the different species, despite the wide variety of parameters and conditions, such as taxonomic identity, number of individuals processed (from 1 for several species up to 30 for *M. martensii*), their sex and the metabolic status of the venom glands, which would have resulted in significant differences in transcript levels for other peptide components of the venom, such as toxins, as reported. This indicates that, while a protracted waiting period between venom extraction and telson dissection and processing may maximize the counts of toxin-coding transcripts, that would not be the case for enzymes.

Recently, a non-lethal method for transcriptomic analysis of the scorpion venom gland has been developed [110]. The mRNA is purified directly from the venom, which eliminates the need for telson dissection and allows a real-time study of the expression levels of mRNAs. Using this approach, it may be possible to accurately determine the time scale of expression of the different enzymes in the venom.

## 4. Conclusions

This is the first time a comprehensive analysis of the enzymes present in scorpion venom is performed. The number of identified enzymes more than quadrupled the number of previously known venom enzymes. Novel enzymes, which can play different roles in envenomation, either through direct toxicity or as spreading factors, venom maturation and preservation, or in prey pre-digestion, were described and annotated.

A venom enzymatic core, constituted by 24 different enzymes determined to be present in all studied scorpion species, was established. This enzymatic core will expand to contain more enzymes, as more transcriptomic and proteomic data become available.

There were several discovered enzymes not previously reported in any other animal venom, with activities that could not be associated with known venom functions. They will require specific biochemical analysis to infer their functions in the scorpion venom.

## 5. Materials and Methods

### 5.1. Transcriptomes Used in the Study

Sequencing reads generated on the Illumina^®^ platform from scorpion venom glands of specimens belonging to 6 of the 20 currently reported families were used [111]. From family Buthidae, the included species were *Centruroides limpidus* (ERR3289195, ERR3289194) [23], *Centruroides hirsutipalpus* (PRJEB34835) [24], *Mesobuthus martensii* (SRR4188636), *Centruroides hentzi* (SRR6041834, SRR6041835) [22] and different ontogenetic stages of *Centruroides vittatus* (SRR5345824, SRR5338072) [25]. From family Hadruridae, the included species were *Hoffmannihadrurus aztecus* (ERR3534794) [20], *Hadrurus spadix* (SRR4069278) [27] and *Hadrurus concolorus* (ERR3561754) [20]. From family Vaejovidae, species *Paravaejovis schwenkmeyeri* (ERR2653951) [29], *Serradigitus gertschi* (ERR2843384) [30] and *Thorellius atrox* (ERR2367086) [31]. For the remaining families, a single representative species was included: *Megacormus gerstchi* (SRR3657526) from family Euscorpiidae [28], *Superstitionia donensis* (SRR4381683) from family Superstitionidae [21] and lastly, *Urodacus yaschenkoi* (SRR1557168) from family Urodacidae [32]. Additionally, for the purpose of comparing expression levels between venom gland and other tissues, the transcriptome from the whole body of *S. donensis* (SRR1721951) was assembled and transcript levels were quantitated.

### 5.2. Read Filtering and De Novo Transcriptome Assembly

Cleaning of the readings was performed using fastp v0.21.0 [112], including removal of adapter sequences and poly(G) tails. Readings with final length greater than or equal to 25 bases, and with Phred quality scores Q greater than or equal to 17 (98% base call accuracy) were retained. The reads were assembled *de novo* with Trinity v2.11.0 [113] using previously-reported parameters [31] and adjusting to 150 the normalized read coverage (—normalize_max_read_cov).

### 5.3. Identification of Transcript Sequences and Putative Coding for Enzymes

Nucleotide sequences were translated into amino acid sequences with the *LongOrfs* function of Transdecoder v5.5.0 [113]. Enzyme identification was performed with the blastx option of diamond v2.0.13.151, using the Braunschweig Enzyme Database (BRENDA, accessed on 1 November 2021) [114] and an e-value of 10^−20^. The resulting enzyme sequences were filtered using UniProt’s metadata to ensure they had an associated signal peptide, had no transmembrane regions and the subcellular location was extracellular. Redundant sequences were removed using CD-HIT v4.8.1 [115].

### 5.4. Identification, Assignment and Annotation of Orthogroups

Sequences sharing orthology relationships were clustered into orthogroups following the methodology previously described [48]. The selected orthogroups contain orthologs present in at least two transcriptomes of different species. Ortholog BRENDA ID assignment was used for orthogroup annotation. Additionally, the presence of signal peptide in the sequences was assessed with SignalP v5 [116], and for each annotation, the cellular location was predicted with DeepLoc v1.0 [117]. Orthogroups that were matched to reference sequences included in the BRENDA/UniProt databases, but resulted in multiple EC assignments, generated conflicts during the sequence annotation process, and were therefore not considered in the analysis.

### 5.5. Identification of Enzymes in the Venom

Transcriptome-derived enzyme sequences were used as templates for cross-matching with the MS/MS spectra generated from the tryptic-digested venoms of 7 species belonging to 4 of the 20 families currently reported [111]. The species were the following: from family Buthidae, *C. limpidus* [23] and *C. hirsutipalpus* [24]; from Vaejovidae, *P. schwenkmeyeri* [29], *S. gertschi* [30] and *T. atrox* [31]; from Euscorpiidae, *M. gertschi* [28]; and from Superstitionidae, *S. donensis* [21]. Proteomic identification was performed with the Proteome Discoverer v1.4 software (Thermo) using the Sequest search engine. The amino acid sequences derived from the transcriptomic analysis of the 14 scorpion species that comply with all the imposed restrictions indicated in Section 5.3 and Section 5.4, were used as a template database to search for coincidences in the 7 available proteomes. The same search parameters were used as in the proteomic analyses mentioned above for the individual species. Enzymes with at least 2 matching peptide fragments with an Xcorr value greater than 2 were considered positive identifications.

### 5.6. RNA-seq Quantification

Estimation of expression levels was carried out with Salmon 1.4.0 [118] for each transcriptome. The transcript expression levels, expressed as transcripts per million (TPM), were normalized with the TPM of the housekeeping phosphoglycerate kinase 1 (PGK1) transcript. A Kruskal–Wallis test was performed to compare the profiles of venom gland enzyme expression levels between the different scorpion species and also between the body and venom gland of *S. donensis*. *p* values < 0.01 were considered statistically significant.

## Figures and Tables

**Table 1:** Enzymes originally reported in the transcriptomic and proteomic analyses on which this study is based. ND stands for Not Determined.

Species (ID)	Year	Transcriptome	Proteome	Distribution	Enzymatic Activity	Reference
*C. hentzi*	2018	Hydrolases Oxidoreductases	Hydrolases Oxidoreductases	North America	ND	[22]
*C. limpidus*	2019	Hydrolases	Hydrolases Transferases	North America	Protease Hyaluronidase	[23]
*C. hirsutipalpus*	2020	Hydrolases Oxidoreductases	Hydrolases Oxidoreductases	North America	ND	[24]
*C. vittatus*	2017	Hydrolases	ND	North America	ND	[25]
*M. martensii*	2021	Hydrolases	ND	East Asia	Hyaluronidase	[26]
*H. aztecus*	2019	Hydrolases	ND	North America	ND	[20]
*H. spadix*	2017	Hydrolases Oxidoreductases Transferases	Hydrolases	North America	ND	[27]
*H. concolorus*	2019	Hydrolases	ND	North America	ND	[20]
*M. gerstchi*	2017	Hydrolases	Hydrolases	Mexico	Phospholipase	[28]
*S. donensis*	2016	Hydrolases	Hydrolases	North America	ND	[21]
*P. schwenkmeyeri*	2018	Hydrolases	Hydrolases	Mexico	Protease Phospholipase Hyaluronidase	[29]
*S. gertschi*	2018	Hydrolases	Hydrolases	North America	Hyaluronidase Phospholipase Gelatinolytic	[30]
*T. atrox*	2017	Hydrolases	Hydrolases	Mexico	ND	[31]
*U. yaschenkoi*	2015	Hydrolases Transferases	ND	Australia	ND	[32]

## Data Availability

Not applicable.

## Supplementary Materials

The following supporting information can be downloaded at: https://www.mdpi.com/article/10.3390/toxins14040248/s1, Table S1: Sequencing and assembly statistics for transcriptomes used in the study, Table S2: ECs identified in the transcriptomic analysis, Table S3: Enzymes found in the analyzed proteomes, Table S4: Enzymes of the scorpion venom enzymatic core, Table S5: Shared ECs among venomous animals.
