# Supplementary material for: The Enzymatic Core of Scorpion Venoms
Source: Toxins (Basel). 2022 Mar 31;14(4):248. doi: 10.3390/toxins14040248 (PMC9030722; doi:10.3390/toxins14040248)
Supplement: Supplementary file 1 [file toxins-14-00248-s001.zip › toxins-1633352-supplementary.pdf]

toxins

MDPI

Article

The Enzymatic Core of Scorpion Venoms

Gustavo Delgado-Prudencio, Jimena I. Cid-Uribe, J. Alejandro Morales, Lourival D. Possani, Ernesto Ortiz and Teresa Romero-Gutiérrez

Table S1. Sequencing and assembly statistics for transcriptomes used in the study.

Scorpion	Number of raw reads (million reads)	Reads after trimming (million reads)	Number of transcripts	Mapping rate (%)	Predicted enzymes
C. hentzi	78.0205	73.0983	628,148	97.4	423
C. limpidus	159.4624	156.8892	215,679	98.4	391
C. hirsutipalpus	5.1071	4.9905	40,254	95.2	141
C. vittatus	96.9241	84.7704	474,383	98.5	359
M. martensii	71.4729	66.2983	325,564	94.7	294
H. aztecus	91.9143	89.3327	90,492	98.0	210
H. spadix	48.4864	46.4710	569,564	96.6	343
H. concolorus	89.4410	88.7126	126,768	97.9	306
P. schwenkmeyeri	17.7176	16.9423	91,166	91.7	250
S. gertschi	17.3635	16.6613	97,022	89.6	244
T. atrox	88.0997	87.0252	136,079	97.7	314
M. gertschi	78.9934	77.8045	112,706	97.7	328
S. donensis	8.3464	8.0281	52,897	91.1	175
U. yaschenkoi	83.8129	78.9262	659,590	97.1	369

Table S2. ECs identified in the transcriptomic analysis.

EC number	Enzyme name	Number of sequences	Number of orthogroups	Found in scorpion proteomes?	Found in other venomous animals? (class)	Found in other venomous animals? (common name)	Presence in scorpion families H|B|V|E|S|U *+	Number of scorpion families with the enzyme
**EC1. Oxidoreductases**								
1.11.1.7	Peroxidase	655	51	Yes	Insecta	bee	1|1|1|1|1|1	6
1.14.17.1	Dopamine beta-monooxygenase	27	2	Yes	No	N/A	1|1|1|1|1|1	6
1.14.17.3	Peptidylglycine monooxygenase (PHM)	16	1	Yes	Arachnida	scorpion, spider	1|1|1|1|1|1	6
1.4.3.13	Protein-lysine 6-oxidase	39	3	No	Lepidosauria	snake	1|1|1|1|1|1	6
1.4.3.2	L-amino-acid oxidase	9	2	No	Lepidosauria	snake	1|1|1|0|1|1	5
1.15.1.1	Superoxide dismutase	24	2	No	Insecta	ant, bee	1|1|1|1|1|1	6
1.11.1.24	Thioredoxin-dependent peroxiredoxin	51	4	No	Lepidosauria	snake	1|1|1|1|1|1	6
**EC2. Transferases**								
**EC number**	**Enzyme name**	**Number of sequences**	**Number of orthogroups**	**Found in scorpion proteomes?**	**Found in other venomous animals? (class)**	**Found in other venomous animals? (common name)**	**Presence in scorpion families H|B|V|E|S|U *+**	**Number of scorpion families with the enzyme**
2.3.2.13	Protein-glutamine gamma-glutamyltransferase	84	4	Yes	No	N/A	1|1|1|1|1|1	6
2.3.2.5	Glutaminyl-peptide cyclotransferase	32	1	No	Lepidosauria	snake	1|1|1|1|1|1	6
2.7.10.1	Receptor protein-tyrosine kinase	1009	62	No	No	N/A	1|1|1|1|1|1	6
**EC3. Hydrolases**								
**EC number**	**Enzyme name**	**Number of sequences**	**Number of orthogroups**	**Found in scorpion proteomes?**	**Found in other venomous animals? (class)**	**Found in other venomous animals? (common name)**	**Presence in scorpion families H|B|V|E|S|U *+**	**Number of scorpion families with the enzyme**
3.1.1.26	Galactolipase	85	6	Yes	No	N/A	1|1|1|1|1|1	6
3.1.1.3	Triacylglycerol lipase	32	4	No	Lepidosauria	snake	1|1|1|1|0|1	5

<table>
<tr><td>3.1.1.34</td><td>Lipoprotein lipase</td><td>4</td><td>3</td><td>No</td><td>Arachnida, Lepidosauria</td><td>scorpion, snake</td><td>0|0|1|0|0|1</td><td>2</td></tr>
<tr><td>3.1.1.4</td><td>Phospholipase A2</td><td>135</td><td>10</td><td>Yes</td><td>Arachnida, Asteroidea, Chilopoda, Gastropoda, Insecta, Lepidosauria</td><td>scorpion, starfish, scolopendra, snail, bee, hornet, snake, heloderma</td><td>1|1|1|1|1|1</td><td>6</td></tr>
<tr><td>3.1.1.7</td><td>Acetylcholinesterase</td><td>53</td><td>8</td><td>Yes</td><td>Arachnida, Lepidosauria</td><td>spider, scorpion, snake</td><td>1|1|1|1|1|1</td><td>6</td></tr>
<tr><td>3.1.1.8</td><td>Cholinesterase</td><td>102</td><td>9</td><td>No</td><td>No</td><td>N/A</td><td>1|1|1|1|1|1</td><td>6</td></tr>
<tr><td>3.1.15.1</td><td>Venom exonuclease 1</td><td>21</td><td>1</td><td>No</td><td>Lepidosauria</td><td>snake</td><td>1|1|1|1|1|1</td><td>6</td></tr>
<tr><td>3.1.22.1</td><td>Deoxyribonuclease II</td><td>14</td><td>1</td><td>No</td><td>Asteroidea, Lepidosauria</td><td>starfish, snake</td><td>1|1|1|1|1|1</td><td>6</td></tr>
<tr><td>3.1.3.2</td><td>Acid phosphatase</td><td>76</td><td>4</td><td>Yes</td><td>Insecta</td><td>bee</td><td>1|1|1|1|1|1</td><td>6</td></tr>
<tr><td>3.1.3.5</td><td>5'-nucleotidase</td><td>2</td><td>2</td><td>Yes</td><td>Arachnida, Lepidosauria</td><td>scorpion, snake</td><td>0|1|0|0|0|0</td><td>1</td></tr>
<tr><td>3.1.4.4</td><td>Phospholipase D</td><td>2</td><td>1</td><td>No</td><td>Arachnida, Lepidosauria</td><td>spider, snake</td><td>1|1|0|0|0|0</td><td>2</td></tr>
<tr><td>3.1.4.41</td><td>Sphingomyelin phosphodiesterase D</td><td>19</td><td>1</td><td>No</td><td>Arachnida</td><td>spider</td><td>1|1|1|1|0|1</td><td>5</td></tr>
<tr><td>3.2.1.1</td><td>Alpha-amylase</td><td>57</td><td>2</td><td>Yes</td><td>Arachnida</td><td>scorpion</td><td>1|1|1|1|1|1</td><td>6</td></tr>
<tr><td>3.2.1.14</td><td>Chitinase</td><td>131</td><td>9</td><td>Yes</td><td>Arachnida, Insecta</td><td>spider, scorpion, wasp</td><td>1|1|1|1|1|1</td><td>6</td></tr>
<tr><td>3.2.1.17</td><td>Lysozyme</td><td>62</td><td>3</td><td>No</td><td>Arachnida</td><td>scorpion</td><td>1|1|1|1|1|1</td><td>6</td></tr>
<tr><td>3.2.1.22</td><td>Alpha-galactosidase</td><td>2</td><td>1</td><td>No</td><td>Insecta</td><td>ant</td><td>0|1|0|0|0|0</td><td>1</td></tr>
<tr><td>3.2.1.35</td><td>Hyaluronoglucosaminidase</td><td>54</td><td>2</td><td>Yes</td><td>Arachnida, Gastropoda, Insecta, Lepidosauria</td><td>scorpion, snail, bee, hornet, wasp, snake</td><td>1|1|1|1|1|1</td><td>6</td></tr>
<tr><td>3.4.11.6</td><td>Aminopeptidase B</td><td>19</td><td>2</td><td>No</td><td>Lepidosauria</td><td>snake</td><td>1|1|1|1|1|1</td><td>6</td></tr>
<tr><td>3.4.15.1</td><td>Peptidyl-dipeptidase A</td><td>107</td><td>11</td><td>Yes</td><td>Actinopterygii, Arachnida, Gastropoda</td><td>fish, scorpion, snail</td><td>1|1|1|1|1|1</td><td>6</td></tr>
</table>

3.4.17.2	Carboxypeptidase B	6	1	No	Lepidosauria	snake	0|1|1|0|0|1	3
3.4.17.10	Carboxypeptidase E	45	4	Yes	No	N/A	1|1|1|1|1|1	6
3.4.17.23	Angiotensin-converting enzyme 2	3	3	Yes	Insecta	ant, bee	1|0|0|1|0|0	2
3.4.17.3	Lysine carboxypeptidase	31	2	No	Arachnida	scorpion	1|1|1|1|1|1	6
3.4.21.1	Chymotrypsin	33	5	No	Arachnida, Insecta	scorpion, ant, hornet	1|1|1|1|1|0	5
3.4.21.34	Plasma Kallikrein	87	25	Yes	No	N/A	1|1|1|1|1|1	6
3.4.21.22	Coagulation factor IXa	5	1	No	Amphibia, Lepidosauria	frog, snake	1|1|0|0|0|0	2
3.4.21.36	Pancreatic elastase	2	2	Yes	No	N/A	1|1|0|0|0|0	2
3.4.21.4	Trypsin	6	5	No	Asteroidea, Chilopoda, Lepidosauria	starfish, scolopendra, snake	0|1|1|1|0|0	2
3.4.21.59	Tryptase	32	12	No	No	N/A	1|1|1|1|1|1	6
3.4.21.6	Coagulation factor Xa	39	4	No	Lepidosauria	snake	1|1|1|1|1|1	6
3.4.21.69	Protein C (activated)	17	7	No	No	N/A	1|1|1|1|0|1	5
3.4.21.79	Granzyme B	3	1	Yes	No	N/A	0|0|1|0|1|0	2
3.4.21.B5	Mast cell protease 5	2	1	No	No	N/A	1|1|0|0|0|0	2
3.4.21.B6	Prostasin	52	15	Yes	No	N/A	1|1|1|1|1|1	6
3.4.21.B60	Epitheliasin	31	9	Yes	Arachnida, Lepidosauria, Mammalia	spider, snake, platypus	1|1|1|1|1|1	6
3.4.21.94	Proprotein convertase 2	29	3	No	Arachnida	scorpion	1|1|1|1|1|1	6
3.4.21.120	Oviductin	11	5	No	Amphibia	frog, toad	1|1|1|0|0|1	4
3.4.22.B49	Cathepsin L1	6	3	No	Arachnida, Chilopoda, Lepidosauria	scorpion, scolopendra, snake	0|1|1|1|0|1	4
3.4.23.3	Gastricsin	5	1	No	No	N/A	1|0|1|1|0|0	3
3.4.24.19	Procollagen C-endopeptidase	121	20	No	No	N/A	1|1|1|1|1|1	6
3.4.24.21	Astacin	26	2	No	Arachnida, Gastropoda, Lepidosauria	scorpion, spider, snail, snake	1|1|1|1|0|1	5

3.4.24.24	Gelatinase A	8	1	No	Amphibia, Arachnida, Lepidosauria	frog, scorpion, snake	1I1I1I0I0I1	4
3.4.24.56	Insulysin	19	3	No	No	N/A	1I1I1I1I1I1	6
3.4.24.63	Meprin B	3	1	No	No	N/A	0I1I0I0I0I0	1
3.4.24.72	Fibrolase	2	1	No	Lepidosauria	snake	0I1I0I0I0I0	1
3.4.24.82	ADAMTS-4 endopeptidase	10	6	No	Lepidosauria	snake	1I1I1I0I0I1	4
3.4.24.87	ADAMTS13 endopeptidase	17	5	No	Lepidosauria	snake	1I1I1I1I1I1	6
3.4.24.B14	Neprilysin-2	344	26	Yes	Arachnida, Insecta, Lepidosauria	scorpion, spider, wasp, snake	1I1I1I1I1I1	6
3.5.1.12	Biotinidase	51	5	No	No	N/A	1I1I1I1I1I1	6
3.5.1.23	Ceramidase	49	2	No	No	N/A	1I1I1I1I1I1	6
3.6.1.5	Apyrase	52	4	Yes	Lepidosauria	snake	1I1I1I1I1I1	6
				**EC4. Lyases**				
**EC number**	**Enzyme name**	**Number of sequences**	**Number of orthogroups**	**Found in scorpion proteomes?**	**Found in other venomous animals? (class)**	**Found in other venomous animals? (common name)**	**Presence in scorpion families HIBIVIEISIU *+**	**Number of scorpion families with the enzyme**
4.2.1.1	Carbonic anhydrase	69	8	No	Arachnida, Hidrozoa, Lepidosauria	spider, hydra, snake	1I1I1I1I1I1	6
4.3.2.5	Peptidyl-alpha-hydroxyglycine alpha-amidating lyase	6	2	No	Arachnida	scorpion	0I0I1I1I0I1	3

* Scorpion families: HadruridaeIButhidaeIVaejovidaeIEuscorpiidaeISuperstitionidaeIUrodacidae (HIBIVIEISIU).

\+ Presence = 1, Absence = 0.

Table S3. Enzymes found in the analyzed proteomes.

EC number	Scorpion species	Score	Coverage (%)	No. peptides	Unique peptides	Theoretical MW (KDa)
3.2.3.35	*C.hi*	203.22	36.66	9	9	46.4
3.4.21.B60	*C.hi*	38.31	9.51	2	2	32.2
1.14.17.3	*C.li*	94.54	10.00	2	2	45.2
2.3.2.13	*C.li*	51.57	5.98	2	2	85.6
3.1.3.2	*C.li*	12.45	16.00	3	1	45.8
3.2.1.1	*C.li*	32.16	14.91	4	4	60.1
3.2.1.35	*C.li*	1695.73	49.38	13	13	46.5
3.4.15.1	*C.li*	25.21	4.79	2	2	71.5
3.4.15.1	*C.li*	11.09	3.61	2	1	94.7
3.4.17.10	*C.li*	17.31	8.69	2	2	53.8
3.4.21.B60	*C.li*	213.32	18.68	2	2	29
3.1.1.4	*M.ge*	138.62	23.59	3	3	21.9
3.2.1.35	*M.ge*	33.46	7.29	2	2	46.1
3.4.15.1	*M.ge*	500.2	42.45	14	13	72
3.4.15.1	*M.ge*	131.56	18.65	6	6	70.5
3.4.15.1	*M.ge*	120.35	13.87	5	5	71.7
3.4.15.1	*M.ge*	25.02	4.24	2	2	74.4
3.4.17.23	*M.ge*	141.62	51.28	3	3	8.9
3.4.21.B6	*M.ge*	102.38	21.77	4	4	32.5
3.4.24.B14	*M.ge*	29.17	9.62	4	4	92.8
3.6.1.5	*M.ge*	242.16	40.00	11	11	63.4
3.4.15.1	*P.sc*	114.96	26.49	7	4	68.3
3.4.15.1	*P.sc*	25.93	13.75	4	1	62.5
3.6.1.5	*P.sc*	34.45	12.52	3	2	68
1.11.1.7	*S.do*	16.43	2.68	4	1	454.5
1.14.17.3	*S.do*	21.31	10.79	2	2	38.5

2.4.1.133; 2.4.1.22; 2.4.1.38; 2.4.1.90	*S.do*	13.34	6.67	2	1	48.3
3.1.1.26	*S.do*	54.7	14.34	4	4	65.5
3.1.1.4	*S.do*	201.27	50.68	6	6	16.3
3.1.1.7; 3.5.1.13	*S.do*	77.08	18.78	4	4	60.2
3.1.1.7; 3.5.1.13	*S.do*	9.75	10.29	2	1	60.6
3.2.1.14	*S.do*	61.46	25.19	4	4	44.9
3.4.15.1	*S.do*	148.27	27.24	8	8	71.3
3.4.15.1	*S.do*	117.32	28.47	8	7	67.3
3.4.15.1	*S.do*	81.88	17.58	6	6	71.2
3.4.21.34	*S.do*	177.52	36.49	6	6	31.9
3.4.21.79	*S.do*	49.55	19.11	2	2	18.2
3.4.24.B14	*S.do*	224.57	23.22	9	7	85.1
3.4.24.B14	*S.do*	45.81	14.53	5	2	85.1
3.6.1.5	*S.do*	398.04	50.35	13	13	63.5
3.1.1.4	*S.ge*	82.73	24.14	5	5	29.9
3.6.1.5	*S.ge*	131.35	14.24	6	6	62.8
1.11.1.7	*T.at*	6.36	2.27	2	1	182.4
1.14.17.1	*T.at*	10.86	7.16	2	2	67.1
3.1.1.4	*T.at*	897.06	17.62	4	4	27.5
3.1.1.4	*T.at*	815.37	27.04	6	6	26.2
3.1.1.4	*T.at*	240.83	13.27	2	2	25.6
3.1.1.7	*T.at*	109.88	20.37	6	6	60.3
3.2.1.35	*T.at*	113.46	20.96	5	4	45.7
3.4.15.1	*T.at*	523.63	24.96	9	9	68
3.4.15.1	*T.at*	182.07	4.61	3	3	73.8
3.4.15.1	*T.at*	120.42	15.81	6	4	71.2
3.4.15.1	*T.at*	65.33	10.02	4	4	75.1

3.4.15.1	*T.at*	64.74	13.13	6	5	71.5
3.4.21.34	*T.at*	452.55	18.66	4	4	31.3
3.4.24.B14	*T.at*	31.33	6.43	3	3	84.3
3.6.1.5	*T.at*	799.41	40.56	17	17	62.5

C.hi: C. hirsutipalpus; C.li: C. limpidus.; M.ge: M. gertschi.; P.sc: P. schwenkmeyeri; S.do: S. donensis; S.ge: S. gertschi; T.at: T. atrox.

Table S4. Enzymes of the scorpion venom enzymatic core.

EC number	Enzyme name
1.11.1.24	Peroxiredoxin-4
1.11.1.7	Peroxidase
1.15.1.1	Superoxide dismutase
2.3.2.13	Protein-glutamine gamma-glutamyltransferase
2.3.2.5	Glutaminyl-peptide cyclotransferase
2.7.10.1	Receptor protein-tyrosine kinase
3.1.1.26	Galactolipase
3.1.1.4	Phospholipase A2
3.1.1.7	Acetylcholinesterase
3.1.1.8	Cholinesterase
3.1.3.2	Acid phosphatase
3.2.1.1	Alpha-amylase
3.2.1.14	Chitinase
3.2.1.17	Lysozyme
3.2.1.35	Hyaluronoglucosaminidase
3.4.15.1	Peptidyl-dipeptidase A
3.4.17.10	Carboxypeptidase E
3.4.21.94	Proprotein convertase 2
3.4.21.B60	Epitheliasin
3.4.24.19	Procollagen C-endopeptidase
3.4.24.56	Insulysin
3.4.24.B14	Neprilysin-2
3.6.1.5	Apyrase
4.2.1.1	Carbonic anhydrase

Table S5. Shared ECs among venomous animals.

EC number	Class	Common name
3.1.1.4	Arachnida, Insecta, Chilopoda, Gastropoda, Asteroidea, Lepidosauria	scorpion, bee, hornet, scolopendra, snail, starfish, heloderma, snake
3.2.1.35	Arachnida, Insecta, Gastropoda, Lepidosauria	scorpion, bee, hornet, wasp, snail, snake
3.4.24.21	Arachnida, Gastropoda, Lepidosauria	scorpion, spider, snail, snake
3.4.24.B14	Arachnida, Insecta, Lepidosauria	scorpion, spider, wasp, snake
3.1.1.3	Arachnida, Insecta	scorpion, ant, hornet, wasp
3.4.21.4	Arachnida, Asteroidea, Chilopoda, Lepidosauria	scorpion, starfish, scolopendra, snake
3.4.21.B60	Arachnida, Lepidosauria, mammalia	scorpion, spider, snake, platypus
4.2.1.1	Arachnida, Hydrozoa, Lepidosauria	scorpion, spider, hydra, snake
3.4.15.1	Arachnida, Gastropoda, Actinopterygii	scorpion, snail, fish
3.4.21.1	Arachnida, Insecta	scorpion, ant, hornet
3.4.22.B49	Arachnida, Chilopoda, Lepidosauria	scorpion, scolopendra, snake
3.2.1.14	Arachnida, Insecta	scorpion, spider, bee
3.4.21.120	Arachnida, Amphibia	scorpion, frog, toad
3.4.21.22, 3.4.24.24	Arachnida, Amphibia, Lepidosauria	scorpion, frog, snake
3.1.1.7, 3.1.4.4	Arachnida, Lepidosauria	scorpion, spider, snake
1.15.1.1, 3.4.17.23	Arachnida, Insecta	scorpion, ant, bee
3.2.1.1, 3.1.3.2, 3.1.4.41	Arachnida, Insecta	scorpion, bee
1.14.17.3	Arachnida	scorpion, spider
1.11.1.24, 1.4.3.13, 1.4.3.2, 2.3.2.5, 3.1.1.26, 3.1.15.1, 3.1.3.5, 3.4.11.6, 3.4.17.2, 3.4.21.6, 3.4.24.72, 3.4.24.82, 3.4.24.87, 3.6.1.5	Arachnida, Lepidosauria	scorpion, snake
3.1.22.1	Arachnida, Asteroidea	scorpion, starfish
3.2.1.22	Arachnida, Insecta	scorpion, ant
3.1.1.34, 3.2.1.17, 3.4.17.3, 4.3.2.5, 1.11.1.7, 1.14.17.1, 2.3.2.13, 2.7.10.1, 3.1.1.8, 3.4.17.10, 3.4.21.34, 3.4.21.36, 3.4.21.59, 3.4.21.69, 3.4.21.79, 3.4.21.94, 3.4.21.B5, 3.4.21.B6, 3.4.23.3, 3.4.24.19, 3.4.24.56, 3.4.24.63, 3.5.1.12, 3.5.1.23	Arachnida	scorpion
